# Primary Intracranial Alveolar Soft Part Sarcoma: Systematic Review

**DOI:** 10.12669/pjms.41.13(PINS-NNOS).13472

**Published:** 2025-12

**Authors:** Haseeb Mehmood Qadri, Hasan Saeed, Muhammad Awais Ahmad, Ali Raza, Tariq Imran Khokhar, Muhammad Imran, Kate Drummond, Asif Bashir

**Affiliations:** 1 Haseeb Mehmood Qadri, MBBS. Punjab Institute of Neurosciences, Lahore, Pakistan; 2 Hasan Saeed, MBBS. Shifa International Hospital, Islamabad, Pakistan. Allama Iqbal Medical College, Lahore, Pakistan; 3 Muhammad Awais Ahmad, MBBS. Allama Iqbal Medical College, Lahore, Pakistan; 4 Ali Raza, MBBS. Lahore General Hospital, Lahore, Pakistan; 5 Tariq Imran Khokhar, MBBS, FCPS Neurosurgery. Punjab Institute of Neurosciences, Lahore, Pakistan; 6 Muhammad Imran, MBBS, FCPS Histopathology Allama Iqbal Medical College, Lahore, Pakistan; 7 Kate Drummond, MBBS, MD, Grad Dip Theol, FRACS (Neurosurgeon) Director of Neurosurgery, RMH (The Royal Melbourne Hospital) Head of Central Nervous System Tumours, VCCC, Grattan St, Parkville VIC 3050, Australia; 8 Asif Bashir, MD, FACS, FFAANS (Diplomate American Board of Neurosurgery). Punjab Institute of Neurosciences, Lahore, Pakistan

**Keywords:** Alveolar soft part, Brain neoplasms, Neoplasms, Pakistan, Sarcoma, Unknown primary

## Abstract

**Background and Objective:**

Alveolar soft part sarcoma is a rare malignancy of unknown histiogenesis. Rarely, primary intracranial alveolar soft part sarcoma has been reported. To review the clinical manifestations, progression and management of this neoplasm was the prime objective of this systematic review.

**Methodology:**

A Preferred Reporting Items for Systematic Reviews and Meta-Analyses-compliant review of PubMed Central, Google Scholar, and ScienceDirect identified 18 primary intracranial alveolar soft-part sarcoma cases (eight reports, three series; 2000–2024). Case reports and series providing data on clinical presentation, radiological features, surgical management, histopathological findings, and patient outcomes were included.

**Results:**

Patients were predominantly female 61.1% (11), with a mean age of 23.3 ± 14.2 years. Headache in 50% (9) and papilledema in 33.3% (6) were common presentations. Frontal lobe involvement was most common in 33.3% (6). Magnetic resonance imaging showed iso+hypointense T1 and iso+hyperintense T2 signals in 22.2% (4) each. Meningioma was the leading differential in 27.8% (5). Gross total resection was achieved in 77.8% (14). Transcription Factor E3 immunopositivity occurred in 55.6% (10) of tumors. Average follow-up was 19.9 months.

**Conclusion:**

Surgical resection remains the primary treatment. Transcription Factor E3 immunoreactivity supports diagnosis. Occult extracranial disease should be excluded. Long-term monitoring is essential due to the indolent course of primary intracranial alveolar soft-part sarcoma.

## INTRODUCTION

Alveolar soft part sarcoma (ASPS) is a rare malignancy with unconfirmed cellular origin, unknown natural history and variable presentation.^1^,^2^ It constitutes less than one percent of all soft tissue sarcomas. ASPS was originally described by Christopherson et al. in 1952 as a unique variant of general soft tissue sarcomas.^3^ This neoplasm affects the younger population, predominantly young females, with a peak incidence between 15 and 35 years.^4^ The primary site of ASPS is most commonly the lower extremities, but they can also arise in the upper extremities, trunk, abdomen and head and neck.^5^,^6^

Rarely, primary intracranial ASPS (PIASPS) has been reported and is in the differential diagnosis of intracranial masses with the radiographic characteristics of meningioma.^7^,^8^ In diagnosing alveolar soft part sarcoma, Alveolar soft part sarcoma chromosome region, candidate 1-transcription factor E3 (ASPSCR1-TFE3) or alveolar soft part locus- transcription factor E3 (ASPL-TFE3), a fusion gene, is informative and has been shown to contribute to tumorigenesis.^9^-^11^ Life-long follow up is essential because of the potential for late recurrence.^12^

We summarize the sparse literature on this rare central nervous system (CNS) neoplasm, focusing on origin, diagnosis, management and prognosis of patients diagnosed with PIASPS.

## METHODOLOGY

The study was registered in the International Prospective Register of Systematic Reviews (PROSPERO) with the ID# CRD42023417711. This systematic review adheres to the Preferred Reporting Items for Systematic Reviews and Meta-Analyses (PRISMA) 2020 standards.^13^

The literature was searched in PubMed, Google Scholar and ScienceDirect using the MeSH terms and keywords “intracranial alveolar soft part sarcoma” OR “primary intracranial alveolar soft part sarcoma” OR “intracerebral alveolar soft part sarcoma” OR “meningeal alveolar soft part sarcoma” OR “alveolar soft part sarcoma of calvarium”. English language publications between January 1, 2000 and January 1, 2025 were included.

The selection of studies was based on predefined inclusion and exclusion criteria.

### Inclusion criteria

- Case reports and series reporting PIASPS, without evidence of systemic or extracranial disease.
- Case reports and series reporting clinical presentation, radiological features, surgical management, histopathological findings, and outcomes.

### Exclusion Criteria

- Reviews, editorials, letters, animal studies.
- Case reports or series in languages other than English.
- Studies with poor methodological quality based on the Joanna Briggs Institute (JBI) critical appraisal check list.

**Fig.1:**
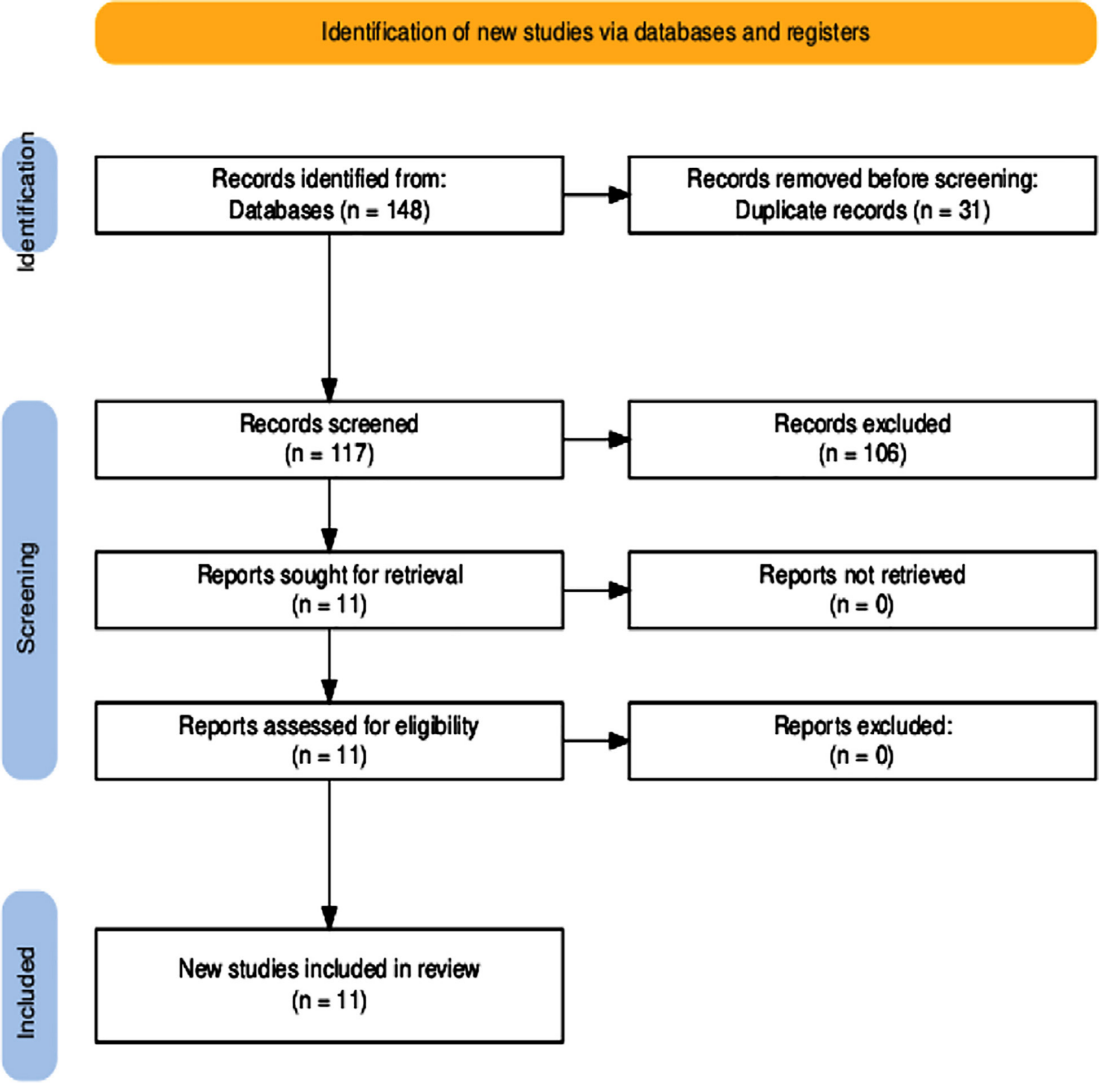
Preferred Reporting Items for Systematic Reviews and Meta-Analyses flow diagram of primary intracranial alveolar soft part sarcoma.

Study selection was carried out by a single author (HMQ). First, titles and abstracts of the studies were identified through the search strategy and were screened against the eligibility criteria. Then, full texts of potentially relevant studies were retrieved and assessed for inclusion. The primary literature search was performed by HMQ and confirmation was done by a Histopathologist (MI). Initially, 148 studies reporting PIASPS between January 1, 2000 and January 1, 2025 were identified. After removal of duplicates, 117 single records were reviewed and 106 reports of ASPS in sites other than the brain or metastatic APSP to the brain were excluded. Finally, eight case reports and three case series were included in the review (Fig.1).

Data extraction was performed using a pre-designed, structured data extraction form, including patient demographics, presenting signs and symptoms, tumor location and laterality, imaging findings, histopathology and immunohistochemistry findings, surgical approaches, postoperative complications, follow-up duration and outcome.

The methodological quality of the included case reports and series was assessed using the JBI critical appraisal tools, tailored to the specific study design (see appendix)^14^,^15^ Based on this assessment, all 11 retrieved studies were included (Table-I).

**Table-I:** Summaries of the included studies in the review.

Study by	Parameters				
	Title	Year	Country of study	Gender	Age (years)
Vinicius et al.^2^	Primary alveolar soft tissue sarcoma of the central nervous system. Case report	2021	Mexico	Male	62
Tao et al.^8^	Primary intracranial alveolar soft-part sarcoma: Report of two cases and a review of the literature	2016	China	Male: 1 Female: 1	20.5 (mean)
Chen et al.^9^	Clinical characteristics and outcomes of primary intracranial alveolar soft-part sarcoma: A case report	2022	China	Male: 2 Female: 3	21 (mean)
Emmez et al.^12^	Primary intracerebral alveolar soft part sarcoma in an 11-year-old girl: Case report and review of the literature	2014	Turkey	Female	11
Kumar et al.^16^	Alveolar soft part sarcoma: the new primary intracranial malignancy	2017	United Kingdom	Male	21
Bodi et al.^17^	Meningeal alveolar soft part sarcoma confirmed by ASPCR1-TFE3 fusion	2008	United Kingdom	Male	39
Ahn et al.^18^	Primary alveolar soft part sarcoma arising from the cerebellopontine angle	2013	South Korea	Female	11
Das et al.^19^	Alveolar soft part sarcoma of the frontal calvarium and adjacent frontal lobe	2012	India	Female	17
Babar et al.^20^	Primary intracranial alveolar soft part sarcoma in 22 years old female: a rare case report with literature review	2022	Pakistan	Female	22
Caporalini et al.^21^	Primary Intracerebral Alveolar Soft Part Sarcoma: Report of a Case and Review of the Literature	2022	Italy	Female	16
Rao et al. 22	Primary Intracranial Alveolar Soft Part Sarcoma: A Report of 3 Cases	2023	India	Male: 1 Female: 2	16.3 (mean)

A narrative synthesis was utilised to summarize and describe the qualitative findings, such as clinical features, histopathological characteristics, and imaging details. For quantitative data, pooled means were calculated for quantitative variables like age and follow-up duration. Percentage distributions were calculated for categorical variables, including gender distribution, symptoms, laterality of lesion, location of the lesion, radiological features of the lesion, histopathology, positive immunohistochemical markers, types of surgical approaches, and patient outcomes.

## RESULTS

Primary intracranial alveolar soft-part sarcoma has a female preponderance. Out of 18, approximately 38.8% (7) cases were males and 61.1% (11) were females. The mean age was 23.3 ± 14.2 years (mean ± standard deviation). Headache in 50% (9) patients, diplopia in 22.2% (4) patients, vomiting in 16.7% (3) patients and limb weakness in 16.7% (3) patients were the most common presenting complaints. The most common signs were papilledema in 33.3% (6) patients, cerebellar signs in 16.7% (3) patients and cranial nerve palsies in 11.1% (2) patients (Table-II). On MRI, the lesions were iso+hypointense on T1-weighted images and iso+hyperintense on T2-weighted images in 22.2% (4) patients (Table-III). PIASPS showed moderate to intense post-contrast enhancement and peritumoral oedema in most of the patients.

**Table-II:** Presenting symptoms and signs (N=18).

Presenting Symptoms	Number of Cases, n	Percentage Occurrence, % (n/N)	Signs at presentation	Number of Cases, n	Percentage Occurrence, % (n/N)
Headache	9	50%	Papilledema	6	33.3%
Diplopia	4	22.2%	Cerebellar signs	3	16.7%
Vomiting	3	16.7%	Cranial nerve palsies	2	11.1%
Limb weakness	3	16.7%	Nystagmus	1	6.0%
Visual disturbances	2	11.1%	Ataxia	1	6.0%
Vision loss	2	11.1%	Exophthalmos	1	6.0%
Tinnitis	2	11.1%	Monoparesis	1	6.0%
Forehead swelling	2	11.1%	Sensorineural hearing loss	1	6.0%

**Table-III:** Radiological findings (N=18).

Radiological findings of the lesion	Number of Cases, n	Percentage Occurrence, % (n/N)
Iso+hypointense on T1WI*	4	22.2%
Iso+hyperintense on T2WI*	4	22.2%
Isointense on T1WI	3	16.7%
Hyperintense on T2WI	3	16.7%
Solid-cystic mass	3	16.7%
Isointense lesion	2	11.1%
Isointense on T2WI	1	6.0%
Hyperdense on CT*	1	6.0%
Heterogeneous on T2WI	1	6.0%
Hypointense on T1WI	1	6.0%
Enhancing intermediate signal on T1WI	1	6.0%

***Abbreviations:*** T1WI: T1 weighted images, T2WI: T2 weighted images, CT: Computed tomography.

The most common initial suspected diagnoses were meningioma in 33.3% (6) and haemangioma in 5.6% (1). The most common sites were frontal lobe in 33.3% (6), parietal lobe in 16.7% (3), parieto-occipital lobe in 11.1% (2) and temporal lobe in 11.1% (2). In terms of laterality 50% (9) patients had tumors on the right side and 33.3% (6) had tumors on the left side of the brain. Surgical approaches were tailored to the site and were most commonly frontal craniotomy in 22.2% (4) and retromastoid suboccipital craniotomy in 11.1% (2). Gross total resection (GTR) was achieved in 77.8% (14) cases. PAS-positive, diastase-resistant cytoplasmic inclusions in 50% (9), clustered polygonal cells in 50% (9) and prominent nucleoli in 50% (9) were the most commonly reported histopathology findings (Table-IV).

**Table-IV:** Histopathological findings of the lesion (N=18).

Histopathological findings of the lesion	Number of Cases, n	Percentage Occurrence, % (n/N)
Periodic Acid-Schiff (PAS) positive, diastase-resistant cytoplasmic inclusions	9	50%
Clustered polygonal cells	9	50%
Prominent nucleoli	9	50%
Alveolar pattern	7	38.9%
Vesicular nuclei	7	38.9%
Clusters lined by thin walled capillaries	7	38.9%
Central dyscohesion	6	33.3%
Granular eosinophilic cytoplasm	4	22.2%
Fibrovascular septae	2	11.1%
Organoid and trabecular pattern	1	6.0%
Abundant mitotic figures	1	6.0%
Homogenous eosinophilic cytoplasm	1	6.0%
Apoptotic bodies	1	6.0%
Rich vascularity	1	6.0%

**Table:** APPENDIX Joanna Briggs Institute Critical Appraisal Checklist for the included case reports

Study by	Q1	Q2	Q3	Q4	Q5	Q6	Q7	Q8	Overall appraisal
Vinicius et al.^2^	Y	Y	Y	Y	Y	Y	Y	Y	Included
Emmez et al.^12^	Y	Y	Y	Y	Y	Y	Y	Y	Included
Kumar et al.^16^	Y	Y	Y	Y	Y	Y	Y	Y	Included
Bodi et al.^17^	Y	Y	Y	Y	Y	Y	Y	Y	Included
Ahn et al.^18^	Y	Y	Y	Y	Y	Y	Y	Y	Included
Das et al.^19^	Y	Y	Y	Y	Y	Y	Y	Y	Included
Babar et al.^20^	Y	Y	Y	Y	Y	Y	Y	Y	Included
Caporalini et al.^21^	Y	Y	Y	Y	Y	Y	Y	U	Included

(Y: Yes, N: No, U: Unclear).

**Table:** Joanna Briggs Institute Critical Appraisal Checklist for the included case series

Study by	Q1	Q2	Q3	Q4	Q5	Q6	Q7	Q8	Q9	Q10	Overall appraisal
Tao et al.^8^	Y	Y	Y	Y	Y	Y	Y	Y	Y	Y	Included
Chen et al.^9^	Y	Y	Y	Y	Y	Y	Y	Y	Y	Y	Included
Rao et al.^22^	Y	Y	Y	Y	Y	Y	Y	Y	Y	Y	Included

(Y: Yes, N: No, U: Unclear).

On immunohistochemistry, strong nuclear positivity of transcription factor E3(TFE3) was reported in 55.6% (10) lesions. Other positive IHC stains were S100 in 27.8% (5) and desmin in 22.2% (4). Proliferation index (Ki-67) was more than 10% in 27.8% (5) neoplasms and less than 10% in 16.7% (3) neoplasms. In 11.1% (2) studies, ASPL-TFE3 fusion product was observed that reported genetic or molecular testing. Adjuvant radiotherapy was given in 55.6% (10) cases with an average dose of 51.6 Gray (Gy). Adjuvant chemotherapy was administered in 22.2% (4) patients, most commonly utilising Pediatric Oncology Group-ifosfamide, carboplatin and etoposide (POG-ICE). Recurrence occurred in 38.9% (7) patients postoperatively. At the last follow-up, 27.8% (5) patients improved, 22.2% (4) remained static while 33.3% (6) succumbed to the disease. Average follow-up duration was 19.9 months.

## DISCUSSION

Primary intracranial alveolar soft-part sarcoma is a rare, slow growing neoplasm with high metastatic potential. A myogenic or a neural crest cell origin is suspected but is still a matter of debate and standard myogenic tissue markers, apart from desmin, have not been detected in this neoplasm.^23^-^25^ Alveolar soft-part sarcoma has the highest incidence of brain metastasis of all soft tissue sarcomas and metastasis may develop prior to primary tumour detection.^16^,^17^ Thus, for intracranial ASPS, any lesion detected extracranially would be regarded as the primary lesion. Naresh et al. hypothesised that the primary tumour remains small and occult due to its non-angiogenic phenotype with poor blood supply but the cells maintain their metastatic potential. Apoptosis and increased turn-over of metastatic cells makes them liable to further mutations, worsening the prognosis.^26^

### Age and gender predilection:

Alveolar soft-part sarcoma shows a characteristic age and sex predilection being the commonest in females below 30 years of age.^18^,^20^-^22^ Our study demonstrated a clear female preponderance for PIASPS.

### Location, signs and symptomatology:

The forebrain is the commonest site for metastatic brain ASPS.^19^ While, PIASPS has been found in various regions of the brain, frontal lobe was the most common in our study 33.3% (6).

In our review, headache in 50% (9), diplopia in 22.2% (4), vomiting in 16.7% (3) and limb weakness in 16.7% (3) were the most common presenting complaints with papilledema in 33.3% (6), cerebellar signs in 16.7% (3) and cranial nerve palsies in 11.1% (2).

### Radiological investigations:

Primary intracranial alveolar soft-part sarcoma mimics meningioma, myeloma, vestibular schwannoma, medulloblastoma and hemangiopericytoma in being iso- to hypointense on T1-weighted images and of variable intensity on T2-weighted images, requiring further workup for definitive diagnosis.^23^,^25^,^27^-^29^ In our review, meningioma and haemangioma were the most common differential diagnoses on imaging.

### Surgical management:

ASPS is a highly vascular neoplasm. Preoperative preparation for large volume haemorrhage is advised.^16^ Gross total resection of the tumour with microscopically clean surgical margins is the key to ensure complete remission. Three out of four patients in our review who underwent sub-total resection had tumour relapse and died, emphasising the importance of complete resection.^8^,^9^,^18^

### Histopathology and IHC:

Primary intracranial alveolar soft-part sarcoma, on standard haematoxylin and eosin (H&E) staining, shows nests of large eosinophilic cells separated by thin fibrovascular septae. These cells contain characteristic PAS-positive, diastase-resistant cytoplasmic inclusions visible on light microscopy.^24^,^26^,^30^ This is in concurrence with our results that showed PAS positive-diastase resistant cytoplasmic inclusions along with clustered polygonal cells and prominent nucleoli. An antibody against the C-terminus of TFE3 has been identified as a highly specific and sensitive marker of ASPS.^31^,^32^ Tanaka et al. described a strong expression of *GPNMB*, a transcriptional target of TFE transcription factors, upregulated by ASPL-TFE3 expression playing an important role in neoplastic invasion, metastases and endothelial migration in the intravascular lesions of ASPS.^33^

The Ki-67 (Kiel-67) proliferation index may be a marker of therapeutic response.^2^ In our review, it was more than 10% in 27.8% (5) patients and less than 10% in 16.7% (3). Higher Ki-67 may also be a biomarker for metastasis.^2^,^27^ Patients with a high Ki-67 index have also been considered suitable for radiotherapy.^9^

### Radiotherapy:

Radiotherapy has been used as an adjuvant and palliative therapy for PIASPS. An average radiation dose of 51.6 Gy has been reported. Portera et al. and Sherman et al. recommended combined radiotherapy and surgery in cases of local recurrence, microscopically positive surgical margins, larger tumor size and difficult surgical location.^25^,^34^ In our study, adjuvant radiotherapy was administered in 55.6% (10) patients. Of these, 50% (5) died, questioning the utility of radiation in managing PIASPS.

### Chemotherapy:

The role of chemotherapy in treatment of PIASPS is unknown^35^,^36^. Extracranial stage IV ASPS has been managed with doxurubicin-based therapy in the past.^35^ Su et al. reported a case of a 24 year old man with primary ASPS of lung and metastases to brain, pancreas and liver. The craniocerebral lesions attained partial remission after whole brain radiotherapy (WBR) and targeted combined immunotherapy (anlotinib and camrelizumab).^36^ Similarly, an anthracycline and ifosfamide containing regimen was used for chemotherapeutic management of soft tissue sarcomas, but such regimens lack efficacy in treating ASPS.^35^ However, Paoluzzi et al. has found a therapeutic sensitivity of ASPS to VEGFR-predominant tyrosine kinase inhibitors (TKIs) (e.g pazopanib).^1^ In our study, 11.1% (2) cases received chemotherapy from paediatric oncology group i.e. ifosfamide, carboplatin and etoposide (POG-ICE). All of them suffered from recurrences or died, indicating the poor efficacy of chemotherapy for PIASPS.

According to Tanaka et al., due to presence of a haemangiopericytic capsule protecting the tumor cells from immune response, long interval between primary diagnosis and systemic metastasis, and decreased permeability of the drugs through the blood-CNS barrier, PIASPS is still considered to be insurmountable to chemotherapeutics.^33^

Over the last decade, treatment options for primary intracranial ASPS have expanded beyond surgery and radiotherapy. While complete excision remains the most important factor for long-term control, more recently, immune checkpoint inhibitors targeting PD-1/PD-L1 have demonstrated encouraging responses, particularly when combined with TKIs, opening the way for multimodal strategies in this rare tumor.^37^

### Follow up and prognosis:

**Fig.2:**
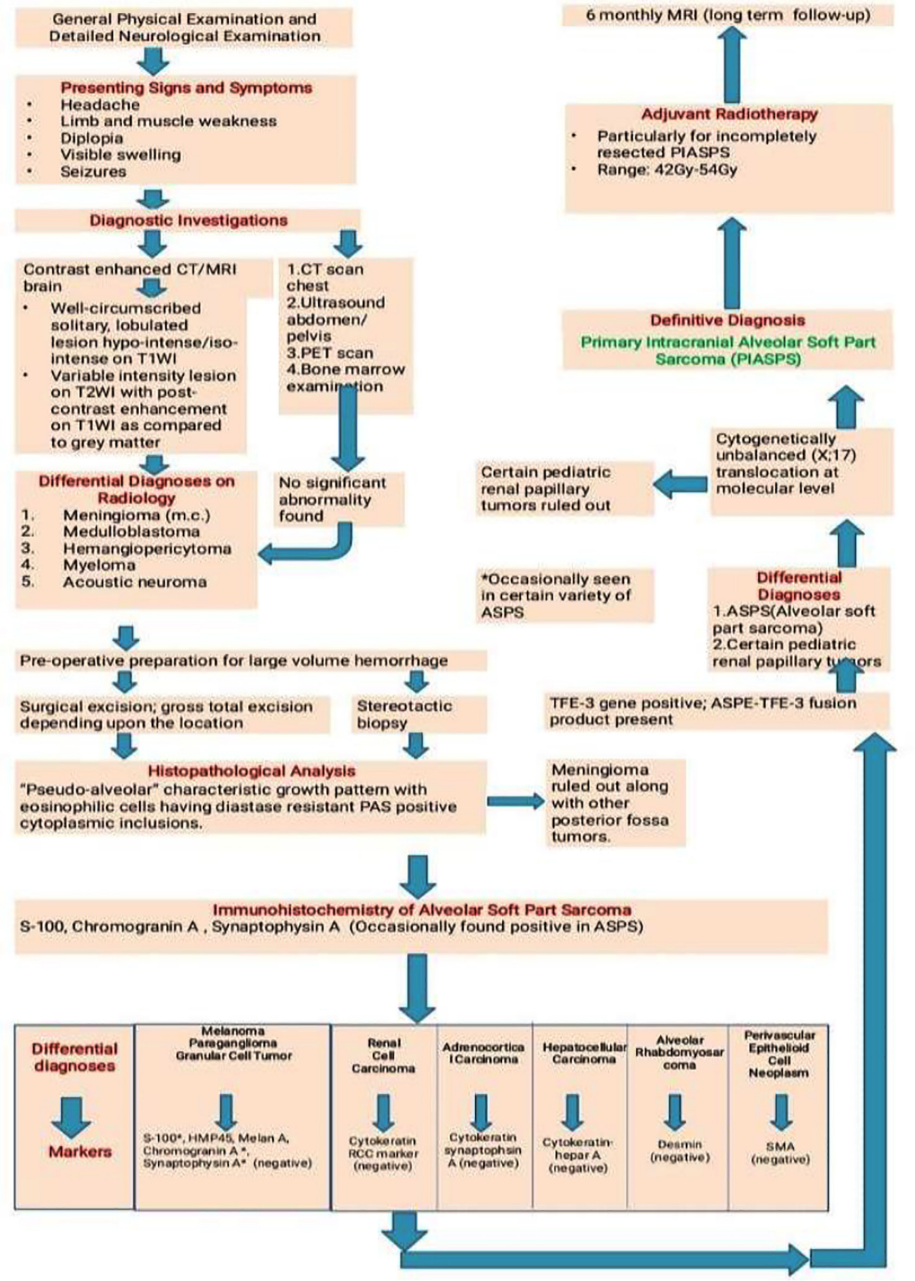
Author-proposed managerial flow diagram of primary intracranial alveolar soft part sarcoma.

Despite slow growth, PIASPS is commonly fatal even with aggressive treatment.^6^ The highly metastatic and relapsing nature of the disease makes long term follow essential. In our study, 38.9% (7) patients experienced postoperative recurrence. On follow-up, 27.8% (5) cases improved, 22.2% (4) remained static while 33.3% (6) died. Author-proposed managerial algorithm for PIASPS from the literature review is given below (Fig.2).

### Limitations:

The results of this review cannot be generalised due to the paucity of literature. Thus, a quantitative meta-analysis was not possible. Many studies have missing surgical and anaesthetic details, as well as grade of neoplasm and follow-up. Immunohistochemical markers and complete molecular diagnostic profile has also not been reported in every included study.

## CONCLUSION

Primary intracranial alveolar soft-part sarcoma is a rare malignancy that resembles meningioma on imaging. Transcription factor E3 antibody is a helpful IHC marker. While direct TFE3 inhibitors are not yet available, targeting the downstream effects of the ASPSCR1::TFE3 fusion presents a promising therapeutic strategy. Ongoing research and clinical trials are essential to validate these approaches and develop effective treatments for ASPS patients. Gross total resection of the tumor accompanied with adjuvant radiotherapy is the currently opted treatment strategy. Long term follow-up for recurrence and metastasis is necessary.

### Clinical Recommendations:

Patients with PIASPS should be treated with gross total surgical resection wherever possible and adjuvant radiotherapy should be considered. They should undergo routine periodic surveillance and long-term follow-up for delayed disease recurrence and metastasis. Further research is required regarding the efficacy of radiotherapy and chemotherapy. Researchers and clinicians interested in PIASPS are encouraged to collaborate internationally to establish such a registry, which could significantly advance understanding and treatment of this rare sarcoma.

### Author`s Contribution:

**HMQ:** Conception, design, acquisition, analysis of data, critically reviewed the manuscript.

**HS:** Analysis and interpretation of data and drafted the manuscript.

**MAA, AA:** Acquisition of data and drafted the manuscript.

**TIK, MI and KD:** Interpretation of data and critically reviewed the manuscript.

**AB:** Interpretation of data, critically reviewed the manuscript, Supervision.

All authors have read the final version to be published and also agreed to be accountable for the integrity of the study.
